# A survey of thermal expansion coefficients for organic molecular crystals in the Cambridge Structural Database

**DOI:** 10.1107/S2052520621003309

**Published:** 2021-05-13

**Authors:** Andrew D. Bond

**Affiliations:** a Yusuf Hamied Department of Chemistry, University of Cambridge, Lensfield Road, Cambridge, CB2 1EW, United Kingdom

**Keywords:** thermal expansion, molecular crystals, Cambridge Structural Database, python API

## Abstract

Typical ranges of thermal expansion coefficients are established for organic molecular crystals in the Cambridge Structural Database. The CSD Python API is used to extract 6201 crystal structures determined close to room temperature and at least one lower temperature down to 90 K. The data set is dominated by structure families with only two temperature points and is subject to various sources of error, including incorrect temperature reporting and missing flags for variable-pressure studies. For structure families comprising four or more temperature points in the range 90–300 K, a linear relationship between unit-cell volume and temperature is shown to be a reasonable approximation. For a selected subset of 210 structures showing an optimal linear fit, the volumetric expansion coefficient at 298 K has mean 173 p.p.m. K^−1^ and standard deviation 47 p.p.m.  K^−1^. The full set of 6201 structures shows a similar distribution, which is fitted by a normal distribution with mean 161 p.p.m. K^−1^ and standard deviation 51 p.p.m. K^−1^, with excess population in the tails mainly comprising unreliable entries. The distribution of principal expansion coefficients, extracted under the assumption of a linear relationship between length and temperature, shows a positive skew and can be approximated by two half normal distributions centred on 33 p.p.m. K^−1^ with standard deviations 40 p.p.m. K^−1^ (lower side) and 56 p.p.m. K^−1^ (upper side). The distribution for the full structure set is comparable to that of the test subset, and the overall frequency of biaxial and uniaxial negative thermal expansion is estimated to be < 5% and ∼30%, respectively. A measure of the expansion anisotropy shows a positively skewed distribution, similar to the principal expansion coefficients themselves, and ranges based on suggested half normal distributions are shown to highlight literature cases of exceptional thermal expansion.

## Abstract 2

Thermal expansion coefficients are calculated for 6201 molecular crystals in the Cambridge Structural Database and the distributions of the values are assessed.

## 1. Introduction   

Structure-property relationships in crystalline materials are of fundamental importance for a huge range of research and practical applications, and the control and design of such properties is at the heart of crystal engineering. For molecular materials, thermal expansion has received some attention in the literature, for example where structures show unusually large or exceptionally anisotropic expansion (Das *et al.*, 2010 ▸; Takahashi & Tamura, 2015 ▸; Alimi *et al.*, 2018 ▸; van der Lee *et al.*, 2018 ▸; Liu *et al.*, 2019 ▸). There have also been some efforts in the crystal engineering literature to link thermal expansion to intermolecular interactions and specific structural features (Bhattacharya & Saha, 2013 ▸; Bhattacharya *et al.*, 2013 ▸; Bhattacharya & Saha, 2014 ▸; Saraswatula *et al.*, 2015 ▸; Hutchins *et al.*, 2016 ▸; Rather & Saha, 2018 ▸; Hutchins *et al.*, 2018*a* ▸; Negi *et al.*, 2018 ▸). The ease and speed by which temperature-dependent X-ray diffraction data can be measured on modern laboratory instruments, combined with accessible analysis tools such as the *PASCal* web server (Cliffe & Goodwin, 2012 ▸), make it straightforward to measure and assess thermal expansion, and there are indications that there is growing interest in reporting such data (*e.g.* Turner *et al.*, 2018 ▸; Brock *et al.*, 2018 ▸; Juneja *et al.*, 2019 ▸; Ding *et al.*, 2020 ▸; Upadhyay *et al.*, 2021 ▸). In this context, a consistent survey of thermal expansion behaviour for the large set of molecular crystals in the Cambridge Structural Database (CSD; Groom *et al.*, 2016 ▸) seems timely.

This paper surveys temperature-dependent data for molecular crystals in the CSD with an aim to establish typical ranges of thermal expansion coefficients and to estimate the frequency of occurrence of interesting features such as uniaxial or biaxial negative thermal expansion (NTE). The survey excludes metal complexes, for which temperature-dependent electronic transitions often influence thermal expansion (*e.g.* Buron Le-Cointe *et al.*, 2012 ▸; Mullaney *et al.*, 2017 ▸), and framework structures (MOFs), for which thermal expansion is highly relevant (*e.g.* Goodwin *et al.*, 2008 ▸; Phillips *et al.*, 2008 ▸; Cliffe *et al.*, 2015 ▸) but often controlled by covalent bonds rather than non-bonded intermolecular interactions. The survey could be extended to such systems, but the generality of the conclusions would be influenced accordingly. This paper focuses specifically on organic molecular crystals.

The nature of the data set, which is subject to various sources of error and comprises principally structures determined at only a few temperature points, necessitates a pragmatic approach by which the expansion is assumed to be linear over the available temperature range. The validity of this assumption is assessed for a targeted subset of reliable structure families before expanding the results to the complete data set.

## 2. Definitions   

The volumetric thermal expansion coefficient is the fractional change in volume per unit change in temperature at constant pressure:
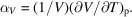
For finite changes under constant pressure over the temperature range Δ*T*, this can be written
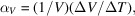
where α_V_ is an average over the range and volume is assumed to change linearly. Coefficients have units of K^−1^ and are commonly quoted in p.p.m. Since Δ*V*/Δ*T* is constant under the linear approximation, α_V_ changes with *V*, and it is necessary to specify a consistent reference point to compare values. The α_V_ values in this study are referenced to the unit-cell volume extrapolated to 298 K.

In general, thermal expansion leads both to changes of length and shape of the crystallographic unit cell, represented overall by a symmetric second-rank tensor. The tensor elements can be derived from unit-cell parameters obtained by variable-temperature diffraction measurements (Schlenker *et al.*, 1978 ▸). The eigenvectors of the tensor form an orthogonal set of principal axes, which are directions of pure length change. The associated eigenvalues give the principal expansion coefficients:
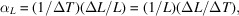
where α_L_ is an average over the temperature range and the length is assumed to change linearly. Again, under the linear assumption, the coefficients change inversely with *L*, and the values in this study are referenced to the length extrapolated to 298 K. For a small expansion, the volumetric expansion coefficient approximates the sum of the three principal expansion coefficients, *i.e.* α_V_ ≃ Σα_L_.

For orthorhombic and higher-symmetry crystal systems, the principal axes are aligned with symmetry axes in the crystal. For the monoclinic crystal system, one axis must be aligned with the twofold symmetry axis but the orientation of the two principal axes in the perpendicular plane is not fixed. For the triclinic crystal system, the principal expansion axes have no fixed alignment with the crystal axes. Since molecular crystals frequently belong to the monoclinic and triclinic systems, the full tensor treatment is necessary to compare expansion coefficients. Several software packages are available to perform the calculations (*e.g.* Angel, 2011 ▸; Langreiter & Kahlenberg, 2015 ▸; Cliffe & Goodwin, 2012 ▸). For integration with the CSD Python API, the calculations in this paper were re-implemented in Python code. Some technical details of the implementation are included in the supporting information.

## 3. Methodology   

The CSD contains a very large number of molecular crystal structures determined over a range of experimental conditions. Multiple crystal structures of the same chemical compound are gathered into families with a common identifier (refcode). Entries within the same refcode family might be polymorphs or redeterminations of the same crystal structure at similar or different conditions. In some cases, exactly the same structure is published more than once. Some work is therefore required to extract a coherent set of structures for analysis. Robust discrimination between polymorphs and re-determinations in the CSD is a non-trivial exercise (van de Streek, 2006 ▸). The task can be simplified here, however, since the large size of the data set means that it is less important to be fully comprehensive. The aim is just to identify a sufficient number of cases where the same structure is confidently established to have been determined at more than one temperature within a chosen range.

Using the Python API, the 1 080 571 entries in the November 2020 CSD release were initially grouped into refcode families. At this stage, the following constraints were applied: (1) entries noted to be measured at non-ambient pressure were discarded; (2) structures without 3-D coordinates were eliminated as a quality-control measure; (3) entries were specified to be organic only; (4) polymeric structures were excluded. Constraints (3) and (4) limit the chemical compounds being considered, within the stated aim to focus on organic molecular crystals. Each refcode family with more than one entry was partitioned into structure families on the basis of similarity between reduced unit-cell parameters, using a simple metric deformation measure (Neumann & Perrin, 2005 ▸). The benefit of using the reduced cell is that it tolerates cases where the same structure may be defined in different space-group settings (*e.g. P*2_1_/*c versus P*2_1_/*n*). Trials in which the space group symbol was also required to match were found to eliminate valid structure families, so this was not applied for the final grouping. Choosing a tolerance to accept a metric match is a compromise between omitting true matches and accepting false matches. A value of 0.12 for the applied deformation measure was found to be suitable by trial-and-error on test cases, although the data set is likely to contain a few cases where valid families have been truncated, or polymorphs with similar metrics have not been distinguished. The reduced cell is also known to be susceptible to edge cases where a subtle change in the lattice can lead to discontinuities, which could lead to false indications of polymorphism, but such cases are likely to be few.

The temperature of each structure determination is recorded in the CSD when it is specified in the literature publication (or associated CIF), else it is assumed to be ‘room temperature’, in the range 283–303 K. Room temperature entries were recorded at 293 K for the subsequent analysis. Each identified structure family was sorted by temperature, then one representative structure was kept at each unique temperature, applying a 10 K tolerance for grouping. For the same structure determined at the same temperature, the entry with the lowest crystallographic *R*-factor was retained. In order to provide a broadly consistent temperature range, the families were finally limited to structures in the range 90–300 K. Any family without a representative at 273 K or above was discarded, and any family for which the total temperature range was less than 50 K was discarded. The final list (available in the supporting information) comprises 6201 unique crystal structures determined close to room temperature and at least one lower temperature down to 90 K. The list comprises 5237 structure families (∼85%) with two entries, 579 families with three entries, 153 families with four entries and 232 families with five or more entries. The largest identified family, {MNPYDO01}^1^, has 14 entries. Chemical diagrams for structure families mentioned in the text are included in the supporting information.

## 4. Results   

### 4.1. Linearity of the volume expansion   

To evaluate the common assumption of linear volume expansion over the temperature range 90–300 K, families were sought with four or more structures and subjected to an unweighted linear least-squares fit of the reduced unit-cell volume against temperature. The *R* ^2^ values for 385 such families indicate that the linear approximation is generally reasonable: ∼50% of the families have *R* ^2^ ≥ 0.980, ∼65% have *R* ^2^ ≥ 0.950 and ∼80% have *R* ^2^ ≥ 0.900. A potential source of error in the data set is highlighted by the first significant outlier in the alphabetical list: {ABELAU} with *R* ^2^ = 0.3729 (see supporting information). The family comprises six structures taken from a single study covering the range 190–290 K (Hutchins *et al.*, 2018*b* ▸), plus one structure listed at 173 K from a separate publication (MacGillivray *et al.*, 2000 ▸). The structure at 173 K has unit-cell volume approximately equal to that at 270 K from the larger study, suggesting that the analysis temperature for ABELAU has probably been reported incorrectly. Removing ABELAU from the family yields a satisfactory linear fit with *R* ^2^ = 0.9633 for the six remaining structures.

To avoid such inconsistencies between different studies, the data subset was narrowed to include only structure families taken from a single publication. This yielded 258 families, for which ∼ 70% have *R* ^2^ ≥ 0.980, ∼85% have *R* ^2^ ≥ 0.950 and ∼90% have *R* ^2^ ≥ 0.900 for the least-squares linear fit of *V* against *T*. Examples of outliers in this data set highlight some additional sources of error:

**{JOGVEJ03}, *R*^2^ = 0.1881 (four structures)**: the family comes from a study of several polymorphs over a range of temperatures (Beldjoudi *et al.*, 2019 ▸). The linear fit is disrupted by JOGVEJ, which is recorded in the CSD at 293 K. Tables in the supporting information of the publication attribute the unit cell of JOGVEJ to 173 K, suggesting that errors have arisen somewhere in the publication/archiving process. Omitting JOGVEJ yields *R* ^2^ = 0.9759 for three remaining structures in the range 195–273 K.

**{MEZKEH08}, *R*^2^ = 0.3843 (four structures)**: the system undergoes an order-disorder phase transition around 225 K (Budzianowski & Katrusiak, 2002 ▸). The first structure in the set, MEZKEH08, is reported at 225 K, and its outlying unit-cell volume seems likely to be influenced by its proximity to the phase transition. Omitting MEZKEH08 yields *R* ^2^ = 0.9571 for three data points in the range 250–293 K.

**{TEDAPC21}, *R*^2^ = 0.4694 (six structures)**: TEDAPC at 296 K has unit-cell volume significantly smaller than that of TEDAPC21 at 120 K. The supporting information document of the publication shows that TEDAPC is measured at 1 GPa (Olejniczak *et al.*, 2013 ▸), which is not flagged in the CSD. Omitting TEDAPC yields *R* ^2^ = 0.9949 for five data points in the range 120–212 K.

These examples illustrate the potential for inconsistency in the extracted data set, which arises from a blend of genuine structural features and issues related to reporting and archiving. On the other hand, the exercise also indicates a pragmatic strategy for further analysis. For valid structure sets (not subject to reporting error and not undergoing phase transitions), it is largely reasonable to assume that the unit-cell volume changes linearly with temperature over the range considered, and this condition can be applied as a criterion to eliminate the most obvious outliers. On this basis, the subset of 258 families from a single publication was narrowed down to 210 families showing *R* ^2^ > 0.96 for a linear least-squares fit of *V* against *T*. These 210 families (listed in the supporting information) are considered to constitute a reliable subset from which to establish indicative ranges for the thermal expansion coefficients. The indications from this set are then used to support the analysis of the full data set, which must contain a significant number of outliers that cannot individually be examined in detail.

### 4.2. Volumetric expansion coefficients   

**Figure 1:**
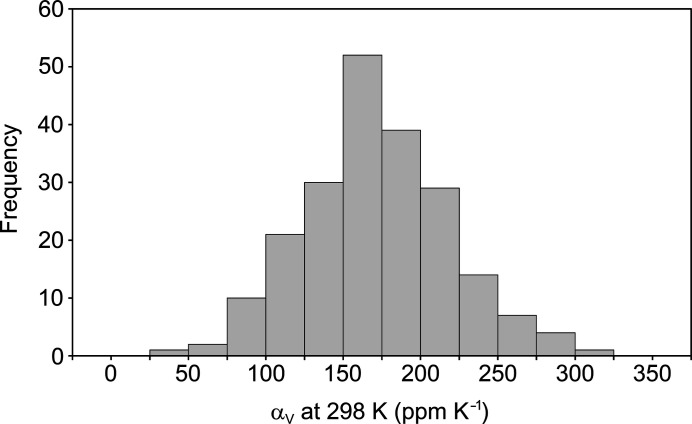
Histogram (bin width 25 p.p.m. K^−1^) of the volumetric expansion coefficient, α_V_, at 298 K for 210 structure families comprising four or more structures from the same publication, with *R* ^2^ > 0.96 for a linear least-squares fit of *V* against *T*.

For the subset of 210 structure families considered to be reliable, the volumetric expansion coefficient was obtained from an unweighted linear least-squares fit of *V* against *T*. The associated standard uncertainty (s.u.) is taken to be the heteroscedasticity-consistent standard error (as suggested by Cliffe & Goodwin, 2012 ▸). The α_V_ values at 298 K for the 210 reliable structure families have mean 173 p.p.m K^−1^ and standard deviation 47 p.p.m. K^−1^, and the derived histogram resembles a normal distribution (Fig. 1 ▸). The largest value in the data set is 301 (20) p.p.m. K^−1^ for 4,4′-di­fluoro­biphenyl ({ZZZAOS03}; Lemée *et al.*, 1987 ▸), although several structure families have similarly high values within the s.u.s estimated on α_V_. Also present near the top of list are {AHEJAZ} (Das *et al.*, 2010 ▸) and {BIJWAS03} (Engel *et al.*, 2014 ▸), both of which have been explicitly reported to show exceptionally large positive volumetric expansion. Hence, the distribution appears to capture expectations from the literature. The smallest value in the data set is 37 (1) p.p.m. K^−1^ for glycylalanine ({GLYALB07}; Capelli *et al.*, 2014 ▸).

**Figure 2:**
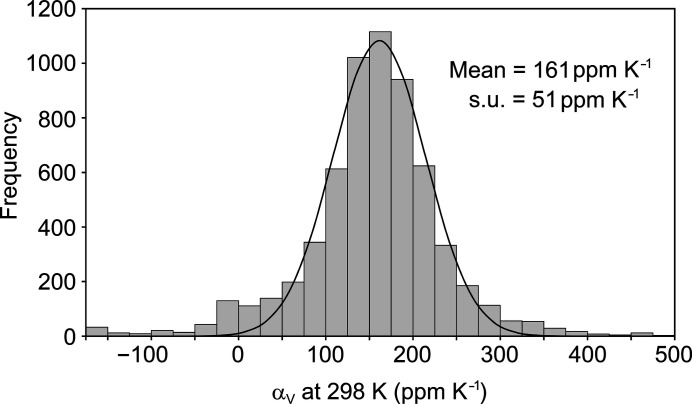
Histogram (bin width 25 p.p.m. K^−1^) of the volumetric expansion coefficient, α_V_, at 298 K derived from 6201 structure families identified in the CSD. The normal distribution is fitted to the histogram values at the mid-temperature of each bin across the full range.

The analysis was then extended to all 6201 identified structure families in the CSD. For families comprising three or more structures, the least-squares linear fit of *V* against *T* was applied as above. For families with only two structures, a simple linear calculation was applied, and s.u.s are not available. The resulting distribution of α_V_ values (Fig. 2 ▸) again resembles a normal distribution, but with excess population in the tails, particularly at the lower end. A normal distribution was fitted to the histogram by minimizing the squared differences between the distribution and histogram values over the full range. This produces the curve shown in Fig. 2 ▸, with mean 161 p.p.m. K^−1^ and standard deviation 51 p.p.m. K^−1^. The similarity to the values obtained for the test subset indicates that the distribution provides reasonable expectations for the volumetric expansion coefficient at 298 K.

The tails of the full α_V_ distribution (Fig. 2 ▸) clearly contain more structure families than captured by the fitted normal distribution. At the upper end, 163 structures (2.5%) have α_V_ greater than the 3σ value (314 p.p.m. K^−1^). Of these, 142 are based on only two temperature points, for which uncertainties cannot be established and which must therefore be viewed with caution. Six of the structures, listed in Table 1 ▸, are based on four or more temperature points. Two of these ({FOCGOT} and {DPANTH04}) show relatively poor linear fits and large s.u. values, so appear unreliable. In fact, {FOCGOT} undergoes conformational change in the crystal over the temperature range (Sim, 1987 ▸). The uncertainty for {DPANTH04} probably arises from the fact that all four structures in the family originate from different studies, which means that measurements have been made using different equipment on four different crystals. The remaining entries in Table 1 ▸ show reasonable linear fits and moderate s.u. values, so they appear to be reliable. Hence, these structures are highlighted to be amongst those showing extreme volumetric expansion in the CSD.

At the lower end of the distribution, the excess frequency around zero can be attributed principally to room-temperature measurements erroneously reported at low temperature (as suspected for ABELAU). There are 300 structures that exceed 3σ at the lower end (8 p.p.m. K^−1^) of which 266 appear to show zero or negative volume expansion. The vast majority of these (92%) are based on only two temperature points, and are assumed to be invalid. There are two families showing apparent negative expansion that are based on a more substantial set of temperature points: {TEDAPC21} (six structures), which was noted above to be an un-flagged variable-pressure study, and {MNPYDO01} (14 structures). Inspection of the latter shows that it is skewed by one structure at 296 K (MNPYDO26; Cai *et al.*, 2014 ▸), which is actually determined at 1.58 GPa but again is not recorded as such in the CSD. Removing MNPYDO26 from the family yields an unremarkable α_v_ = 158 (5) p.p.m. K^−1^ for the remaining 13 data points over the range 106–285 K (see supporting information).

### 4.3. Principal expansion coefficients   

For each structure family, the reduced unit-cell parameters were used to construct strain tensors according to the method of Schlenker *et al.* (1978 ▸). The linear Lagrangian tensor was applied.^2^ The strain coefficients, Δ*L*/*L*, extracted as eigenvalues of the strain tensor, are converted to thermal expansion coefficients by dividing by Δ*T*. The method, based on snapshots of the metric at finite temperature intervals, produces average coefficients over the temperature range. For the monoclinic and triclinic crystal systems, the results also represent an average of the orientations of the non-constrained principal axes. For families with three or more data points, each coefficient and associated s.u. was obtained from a linear least-squares fit of Δ*L*/*L* against *T*. For families with only two structures, the coefficients were obtained from the one available strain tensor, and errors cannot be estimated. As for the volumetric coefficient, the reported values are extrapolated to refer to 298 K. For several test cases, the extent of this extrapolation and comparisons to the results from *PASCal* (Cliffe & Goodwin, 2012 ▸) are included in the supporting information.

**Figure 3:**
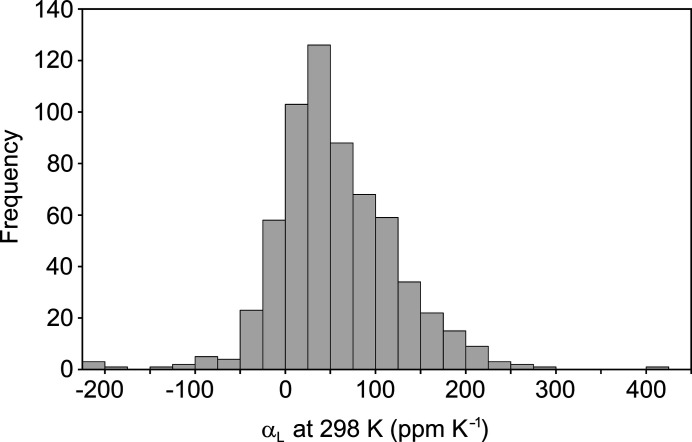
Histogram (bin width 25 p.p.m. K^−1^) of the principal expansion coefficients, α_L_, at 298 K derived from the 210 reliable structure families. The distribution includes all three linear coefficients for each structure family (630 α_L_ values in total).

A histogram of the principal expansion coefficients for the test subset of 210 structure families (Fig. 3 ▸) has its maximum in the 25–50 p.p.m. K^−1^ bin and shows a clear positive skew. The histogram includes all α_L_ values in the 210 families (630 data points in total) since the aim is to compare any α_L_ value in any structure to all α_L_ values in all structures (not necessarily to compare the smallest α_L_ in each structure to the smallest α_L_ in other structures, *etc.*). In the sorted list, three structures stand out as having extreme positive and negative coefficients (Table 2 ▸). {AHEJAZ} (Das *et al.*, 2010 ▸) has been noted above, while {BOQHOE01} has been reported recently to show supercolossal uniaxial NTE (Liu *et al.*, 2019 ▸). Hence, the data set again appears to reflect expectations from the literature. A third entry in Table 1 ▸, {XIWREA07}, undergoes a phase transition in the range 175–220 K (Jackson *et al.*, 2016 ▸), which affects the extracted average values. Amongst the test subset, there are seven structures with two negative principal coefficients (showing biaxial NTE), but only two of these show two substantial negative coefficients (< −10 p.p.m. K^−1^) that appear to be conclusive within the estimated errors. These are {AHEJAZ} (Das *et al.*, 2010 ▸)^3^ and {HACTPH30} (Sztylko *et al.*, 2019 ▸). Thus, biaxial NTE is clearly rare amongst the test set. Uniaxial NTE, on the other hand, is far more common: 83 of the 210 structures display one negative α_L_ value, of which at least 50 (∼25% of the set) appear to be conclusive within the estimated errors on the α_L_ values.

**Figure 4:**
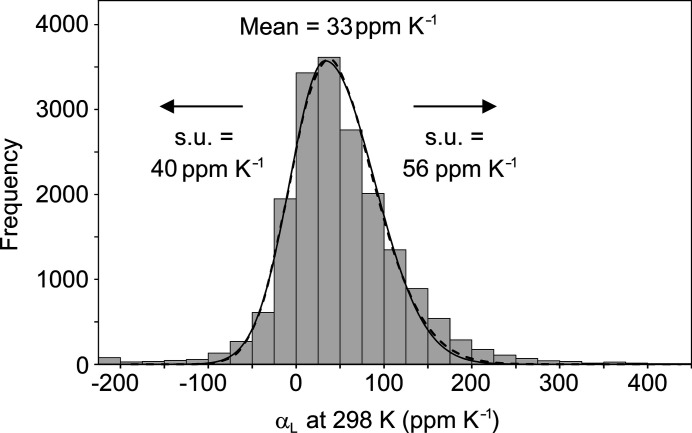
Histogram (bin width 25 p.p.m. K^−1^) of the principal expansion coefficients, α_L_, at 298 K for all 6201 structure families. The distribution includes all three linear coefficients for each structure family (18 603 α_L_ values in total). The dashed line shows a continuous skew normal distribution fitted to the histogram values across the range, approximated by two half normal distributions (solid line).

Extending the study to all 6201 structure families produces the distribution of principal expansion coefficients shown in Fig. 4 ▸. Although it is not possible to assess the uncertainties for the vast majority of the data set, the similarity between the distributions for the total data set and the test subset (Fig. 3 ▸) gives confidence that the extracted principal coefficients are meaningful. The histogram was fitted by a continuous skew normal distribution (see supporting information), which was then approximated by two half normal distributions, centred on 33 p.p.m. K^−1^ with standard deviation 40 p.p.m. K^−1^ (lower side) and 56 p.p.m. K^−1^ (upper side) (Fig. 4 ▸). Hence, the distribution suggests that principal expansion coefficients outside of the approximate range −87 < α_L_ < 201 p.p.m. K^−1^ are exceptional at the 3σ level. The proportions of structures showing apparent biaxial and uniaxial NTE are 5% and 34%, respectively, although the lack of associated errors for the majority of the data set make these values highly uncertain. Comparing to the values seen for the test subset, reasonable estimates applying to all molecular structures in the CSD are suggested to be < 5% for biaxial NTE and ∼30% for uniaxial NTE.

### 4.4. Degree of anisotropy   

**Figure 5:**
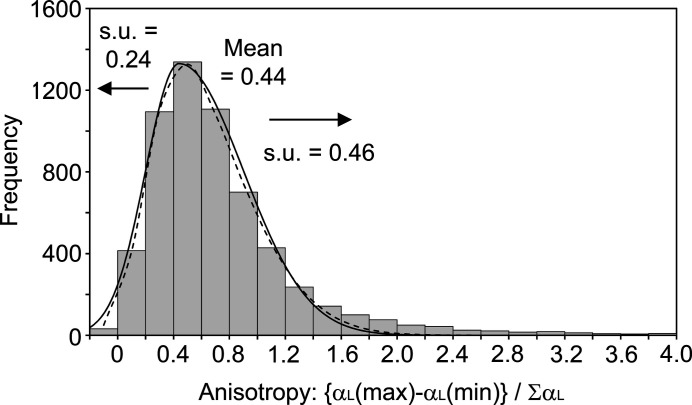
Distribution of the anisotropy measure {α_L_(max) – α_L_(min)}/α_V_ for all 6201 structure families. The dashed line shows a continuous skew normal distribution fitted to the histogram values across the range, approximated by two half normal distributions (solid line).

The degree of anisotropy in the thermal expansion is quantified here as {α_L_(max) – α_L_(min)}/Σα_L_. In the test set, the greatest anisotropy is seen for the three structures listed in Table 1 ▸, with the smallest value (0.02) seen for diiso­propyl­ammonium bromide ({TEJKUO09}; Fu *et al.*, 2013 ▸). For the full data set, the distribution of the anisotropy measure resembles that of the principal coefficients themselves, centred close to 0.5 with a positive skew. The histogram was fitted as described for the principal coefficients, using a continuous skew normal distribution which was then approximated by two half normal distributions, with a common mean of 0.44 and standard deviations of 0.24 (lower side) and 0.46 (upper side) (Fig. 5 ▸). The full data set shows numerous negative anisotropy values that arise from the negative values of α_V_ noted in §4.2, all of which are assumed to be invalid. At the upper end of the distribution, the largest anisotropy values arise from anomalously small volume changes, which are dominated by the peak seen around zero in the volumetric expansion (Fig. 2 ▸) and are again considered to be unreliable. In addition to the cases already highlighted in Table 2 ▸, there are three cases of extreme anisotropy (exceeding that of {AHEJAZ}) based on a good linear fit to several temperature points, which appear to be reliable within the associated s.u.s on the α_L_ values (Table 3 ▸). {JETRIJ} is another example undergoing conformational change in the crystal over the temperature range (Sim, 1990 ▸), {UROBUA10} has been specifically reported to show exceptional thermal expansion properties (van der Lee *et al.*, 2018 ▸), and {RALLAU05} appears to be a reliable example that has not previously been highlighted (Dulani Dhanapala *et al.*, 2017 ▸). Again, it is encouraging that the presented distributions draw attention to these interesting cases.

## 5. Conclusions   

This survey provides an indication of the range of thermal expansion coefficients shown by molecular crystals currently in the Cambridge Structural Database. The values observed for the volumetric expansion coefficient, obtained from a linear fit of *V* against *T*, and extrapolated to 298 K, are described by a normal distribution with mean 161 p.p.m. K^−1^ and standard deviation 51 p.p.m. K^−1^. The values of all extracted principal expansion coefficients, also based on a linear fit of *L* against *T*, are described by two half normal distributions, centred on 33 p.p.m. K^−1^ with standard deviation 40 p.p.m. K^−1^ (lower side) and 56 p.p.m. K^−1^ (upper side). The occurrence of biaxial and uniaxial NTE is estimated to be < 5% and ∼30%, respectively. The linear assumption applied to extract both the volumetric and principal expansion coefficients is a significant approximation, but it is shown to be reasonable over the considered temperature range for a carefully chosen test set of structure families, and it is required to extract any information from the majority of the data set comprising only two temperature points. Clearly, the linear approximation will produce misleading results in cases that do not conform to the linear assumption. In the current data set, this could include structures undergoing phase transitions within the temperature range or those with reporting/archiving errors, and a few such cases are mentioned herein. The influence of such examples on the survey results should be mitigated by the large size of the total data set, and it is shown that the distributions obtained for the complete data set resemble closely those obtained for a more carefully chosen subset. The distributions provide a guide to the potential significance of newly observed thermal expansion behaviour, and the results seem to be largely consistent with exceptional cases in the existing literature. New cases at the extremes of these distributions might therefore be highlighted as exceptional, or identified as targets for further analysis. When comparing to these distributions, it is required to apply the same linear fitting methodology, which is easily accessible through the *PASCAL* web tool (Cliffe & Goodwin, 2012 ▸), for example. Relating the results for the large data set to structural classes or specific structural features is a much more significant exercise, which has not been attempted here.

## Supplementary Material

Lists of refcode families for the full data set (6201) and test data subset (210) (MS Excel format). DOI: 10.1107/S2052520621003309/rm5048sup1.xlsx

Lists of expansion coefficients for the full data set (6201) and test data subset (210) (MS Excel format). DOI: 10.1107/S2052520621003309/rm5048sup2.xlsx

Supporting information file. DOI: 10.1107/S2052520621003309/rm5048sup1.pdf

## Figures and Tables

**Table 1:** Examples of families exceeding the upper 3σ level for the volumetric expansion coefficient (327 p.p.m. K^−1^) amongst the full data set Only families comprising four or more temperature points are listed. The 2-digit suffix on the refcode is listed in parentheses ([] denotes no suffix).

Refcode family	Δ*T* (K)	*R* ^2^ for LS fit	α_V_ (298 K)	References
FOCGOT ([],01,02,03)	127–293	0.8609	371 (76)	Sim (1987 ▸)
DPANTH (04,05,02,01)	113–293	0.8129	362 (119)	Okutsu *et al.* (2005 ▸); Pospiech & Bolte (2011 ▸); Abboud *et al.* (1990 ▸); Choi & Marinkas (1980 ▸)
ZZZKAY (03,01,04,05)	100–293	0.9339	362 (31)	Fabbiani *et al.* (2014 ▸); Usanmaz & Adler (1982 ▸)
METNAM (07,03,05,01)	125–293	0.9873	343 (14)	Filhol *et al.* (1980 ▸); Krebs *et al.* (1979 ▸)
PBPACB (02,03,01,[])	150–293	0.9546	340 (29)	Waddell (2015 ▸); Bolte (2017 ▸); Hosten & Betz (2015 ▸); Lau *et al.* (1976 ▸)
TETROL (02,03,04,05,[])	118–293	0.9950	336 (12)	Saraswatula *et al.* (2015 ▸)

**Table 2:** Extreme values of principal expansion coefficients (p.p.m. K^−1^) identified amongst the subset of 210 reliable structure families The volumetric coefficient and anisotropy measure are also listed. The 2-digit suffices on the refcodes making up the family are listed in parentheses ([] denotes no suffix).

Refcode family	Δ*T* (K)	α_V_	α_L_(1)	α_L_(2)	α_L_(3)	Anisotropy: {α_L_(3)–α_L_(1)}/Σα_L_	Reference
AHEJAZ ([],01,02,03,04,05)	225–300	291 (17)	−249 (34)	−99 (22)	623 (64)	3.163	Das *et al.* (2010 ▸)
BOQHOE01 (01,03,04,02,06)	150–275	271 (7)	−484 (154)	172 (16)	547 (130)	4.369	Liu *et al.* (2019 ▸)
XIWREA07 (07,06,05,04,03,02,01)	100–273	214 (7)	−388 (45)	123 (4)	422 (39)	5.173	Jackson *et al.* (2016 ▸)

**Table 3:** Structure families with four or more temperature points showing good linear fits and anisotropy values exceeding {AHEJAZ} (Table 2 ▸) The 2-digit suffices on the refcodes making up the family are listed in parentheses ([] denotes no suffix).

Refcode family	Δ*T* (K)	α_V_	α_L_(1)	α_L_(2)	α_L_(3)	Anisotropy: {α_L_(3)–α_L_(1)}/Σα_L_	Reference
JETRIJ ([],01,03,02)	130–293	155 (16)	−254 (42)	160 (13)	228 (37)	3.575	Sim (1990 ▸)
UROBUA (10,06,07,05,08,09,[])	175–293	280 (35)	−330 (5)	44 (9)	54 (25)	3.422	van der Lee *et al.* (2018 ▸)
RALLAU (05,04,02,01,[])	100–300	190 (13)	−225 (48)	52 (12)	339 (37)	3.383	Dulani Dhanapala *et al.* (2017 ▸)

## Footnotes

1 Structure families are represented by a refcode enclosed in braces, where the refcode is the first entry (lowest T) in the family. Refcodes without braces refer to individual CSD entries.

2 As discussed by Cliffe & Goodwin (2012 ▸), other strain definitions might be applied but the results generally are not altered substantially. The strain calculations in the new Python code were validated against results from the Bilbao Crystallographic Server (Aroyo *et al.*, 2006 ▸) and the *Win_Strain* program (Angel, 2011 ▸).

3 {AHEJAZ} provides an example of difficulties that can arise when comparing reported values of thermal expansion coefficients. Das *et al.* (2010 ▸) report a range of coefficients between 225 and 330 K, each obtained from the gradient between the lower temperature point and 330 K. A comparison between that approach and the calculations in this paper is provided in the supporting information.

## References

[bb1] Abboud, K. A., Simonsen, S. H. & Roberts, R. M. (1990). *Acta Cryst.* C**46**, 2494–2496.

[bb2] Alimi, L. O., Lama, P., Smith, V. J. & Barbour, L. J. (2018). *CrystEngComm*, **20**, 631–635.

[bb3] Angel, R. J. (2011). *Win_Strain.* http://www.rossangel.com/text_strain.htm.

[bb4] Aroyo, M. I., Perez-Mato, J. M., Capillas, C., Kroumova, E., Ivantchev, S., Madariaga, G., Kirov, A. & Wondratschek, H. (2006). *Z. Kristallogr.* **221**, 15–27.

[bb5] Beldjoudi, Y., Arauzo, A., Campo, J., Gavey, E. L., Pilkington, M., Nascimento, M. A. & Rawson, J. M. (2019). *J. Am. Chem. Soc.* **141**, 6875–6889. 10.1021/jacs.8b11528 30875208

[bb6] Bhattacharya, S. & Saha, B. K. (2013). *Cryst. Growth Des.* **13**, 3299–3302.

[bb7] Bhattacharya, S. & Saha, B. K. (2014). *CrystEngComm*, **16**, 2340–2343.

[bb8] Bhattacharya, S., Saraswatula, V. G. & Saha, B. K. (2013). *Cryst. Growth Des.* **13**, 3651–3656.

[bb9] Bolte, M. (2017). Private communication (refcode PBPACB03). CCDC, Cambridge, England.

[bb10] Brock, A. J., Whittaker, J. J., Powell, J. A., Pfrunder, M. C., Grosjean, A., Parsons, S., McMurtrie, J. C. & Clegg, J. K. (2018). *Angew. Chem. Int. Ed.* **57**, 11325–11328. 10.1002/anie.201806431 29998602

[bb11] Budzianowski, A. & Katrusiak, A. (2002). *Acta Cryst.* B**58**, 125–133. 10.1107/s0108768101017955 11818660

[bb12] Buron-Le Cointe, M., Hébert, J., Baldé, C., Moisan, N., Toupet, L., Guionneau, P., Létard, J. F., Freysz, E., Cailleau, H. & Collet, E. (2012). *Phys. Rev. B*, **85**, 064114.

[bb13] Cai, W., He, J., Li, W. & Katrusiak, A. (2014). *J. Mater. Chem. C*, **2**, 6471–6476.

[bb14] Capelli, S. C., Bürgi, H.-B., Dittrich, B., Grabowsky, S. & Jayatilaka, D. (2014). *IUCrJ*, **1**, 361–379. 10.1107/S2052252514014845 PMC4174878 25295177

[bb15] Choi, C. S. & Marinkas, P. L. (1980). *Acta Cryst.* B**36**, 2491–2493.

[bb16] Cliffe, M. J. & Goodwin, A. L. (2012). *J. Appl. Cryst.* **45**, 1321–1329.

[bb17] Cliffe, M. J., Hill, J. A., Murray, C. A., Coudert, F.-X. & Goodwin, A. L. (2015). *Phys. Chem. Chem. Phys.* **17**, 11586–11592. 10.1039/c5cp01307k 25866163

[bb18] Das, D., Jacobs, T. & Barbour, L. J. (2010). *Nat. Mater.* **9**, 36–39. 10.1038/nmat2583 19935666

[bb19] Ding, X., Unruh, D. K., Groeneman, R. H. & Hutchins, K. M. (2020). *Chem. Sci.* **11**, 7701–7707. 10.1039/d0sc02795b PMC7480503 32953037

[bb20] Dulani Dhanapala, B., Mannino, N. A., Mendoza, L. M., Tauni Dissanayake, K., Martin, P. D., Suescun, L. & Rabuffetti, F. A. (2017). *Dalton Trans.* **46**, 1420–1430. 10.1039/c6dt04152c 28054697

[bb21] Engel, E. R., Smith, V. J., Bezuidenhout, C. X. & Barbour, L. J. (2014). *Chem. Commun.* **50**, 4238–4241. 10.1039/c4cc00849a 24633431

[bb22] Fabbianni, F. P. A., Pulham, C. R. & Warren, J. E. (2014). *Z. Krist. Cryst. Mater.* **229**, 667–675.

[bb23] Filhol, A., Bravic, G., Rey-Lafon, M. & Thomas, M. (1980). *Acta Cryst.* B**36**, 575–586.

[bb24] Fu, D.-W., Cai, H.-L., Liu, Y., Ye, Q., Zhang, W., Zhang, Y., Chen, X.-Y., Giovannetti, G., Capone, M., Li, J. & Xiong, R.-G. (2013). *Science*, **339**, 425–428. 10.1126/science.1229675 23349285

[bb25] Goodwin, A. L., Calleja, M., Conterio, M. J., Dove, M. T., Evans, J. S. O., Keen, D. A., Peters, L. & Tucker, M. G. (2008). *Science*, **319**, 794–797. 10.1126/science.1151442 18258911

[bb26] Groom, C. R., Bruno, I. J., Lightfoot, M. P. & Ward, S. C. (2016). *Acta Cryst.* B**72**, 171–179. 10.1107/S2052520616003954 PMC4822653 27048719

[bb27] Hosten, E. C. & Betz, R. (2015). *Z. Kristallogr. NCS*, **230**, 59–60.

[bb28] Hutchins, K. M., Kummer, K. A., Groeneman, R. H., Reinheimer, E. W., Sinnwell, A. A., Swenson, D. C. & MacGillivray, L. R. (2016). *CrystEngComm*, **18**, 8354–8357.

[bb29] Hutchins, K. M., Unruh, D. K., Carpenter, D. D. & Groeneman, R. H. (2018*a*). *CrystEngComm*, **20**, 7232–7235.

[bb30] Hutchins, K. M., Unruh, D. K. & Groeneman, R. H. (2018*b*). *New J. Chem.* **42**, 16460–16463.

[bb31] Jackson, M. A., Blackburn, J. A., Price, N. P. J., Vermillion, K. E., Peterson, S. C. & Ferrence, G. M. (2016). *Carbohydr. Res.* **432**, 9–16. 10.1016/j.carres.2016.06.003 27341396

[bb32] Juneja, N., Unruh, D. K., Bosch, E., Groeneman, R. H. & Hutchins, K. M. (2019). *New J. Chem.* **43**, 18433–18436.

[bb33] Krebs, B., Mandt, J., Cobbledick, R. E. & Small, R. W. H. (1979). *Acta Cryst.* B**35**, 402–404.

[bb34] Langreiter, T. & Kahlenberg, V. (2015). *Crystals*, **5**, 143–153.

[bb35] Lau, B. W., Yoon, Y. K. & Suh, I.-H. (1976). *J. Korean Phys. Soc.* **9**, 29–37.

[bb36] Lee, A. van der, Roche, G. H., Wantz, G., Moreau, J. J., Dautel, O. J. & Filhol, J.-S. (2018). *Chem. Sci.* **9**, 3948–3956. 10.1039/c8sc00159f PMC5941202 29780527

[bb37] Lemée, M. H., Toupet, L., Délugeard, Y., Messager, J. C. & Cailleau, H. (1987). *Acta Cryst.* B**43**, 466–470.

[bb38] Liu, H., Gutmann, M. J., Stokes, H. T., Campbell, B. J., Evans, I. R. & Evans, J. S. O. (2019). *Chem. Mater.* **31**, 4514–4523.

[bb39] MacGillivray, L. R., Reid, J. L. & Ripmeester, J. A. (2000). *J. Am. Chem. Soc.* **122**, 7817–7818.

[bb40] Mullaney, B. R., Goux-Capes, L., Price, D. J., Chastanet, G., Létard, J. F. & Kepert, C. J. (2017). *Nat. Commun.* **8**, 1053. 10.1038/s41467-017-00776-1 PMC5648752 29051479

[bb41] Negi, L., Shrivastava, A. & Das, D. (2018). *Chem. Commun.* **54**, 10675–10678. 10.1039/c8cc05859h 30137090

[bb42] Neumann, M. A. & Perrin, M. (2005). *J. Phys. Chem. B*, **109**, 15531–15541. 10.1021/jp050121r 16852970

[bb43] Okutsu, T., Isomura, K., Kakinuma, N., Horiuchi, H., Unno, M., Matsumoto, H. & Hiratsuka, H. (2005). *Cryst. Growth Des.* **5**, 461–465.

[bb44] Olejniczak, A., Anioła, M., Szafrański, M., Budzianowski, A. & Katrusiak, A. (2013). *Cryst. Growth Des.* **13**, 2872–2879.

[bb45] Phillips, A. E., Goodwin, A. L., Halder, G. J., Southon, P. D. & Kepert, C. J. (2008). *Angew. Chem. Int. Ed.* **47**, 1396–1399. 10.1002/anie.200704421 18095370

[bb46] Pospiech, S. & Bolte, M. (2011). Private communication (refcode DPANTH05). CCDC, Cambridge, England.

[bb47] Rather, S. A. & Saha, B. K. (2018). *Cryst. Growth Des.* **18**, 2712–2716.

[bb48] Saraswatula, V. G., Bhattacharya, S. & Saha, B. K. (2015). *New J. Chem.* **39**, 3345–3348.

[bb49] Schlenker, J. L., Gibbs, G. V. & Boisen, M. B. (1978). *Acta Cryst.* A**34**, 52–54.

[bb50] Sim, G. A. (1987). *J. Chem. Soc. Chem. Commun.* pp. 1118–1120.

[bb51] Sim, G. A. (1990). *Acta Cryst.* B**46**, 676–682.

[bb52] Streek, J. van de (2006). *Acta Cryst.* B**62**, 567–579.

[bb53] Sztylko, M., Malinska, M., Petricek, V., Gutmann, M. J. & Hoser, A. A. (2019). *Cryst. Growth Des.* **19**, 5132–5141.

[bb54] Takahashi, H. & Tamura, R. (2015). *CrystEngComm*, **17**, 8888–8896.

[bb55] Turner, T. D., Lai, X. & Roberts, K. J. (2018). *CrystEngComm*, **20**, 4099–4102.

[bb56] Upadhyay, P. P, Mishra, M. K., Ramamurty, U. & Bond, A. D. (2021). *CrystEngComm*, **23**, 1226–1233.

[bb57] Usanmaz, A. & Adler, G. (1982). *Acta Cryst.* B**38**, 660–662.

[bb58] Waddell, P. G. (2015). Private communication (refcode PBPACB02). CCDC, Cambridge, England.

