# Supplementary material for: A survey of thermal expansion coefficients for organic molecular crystals in the Cambridge Structural Database
Source: Acta Crystallogr B Struct Sci Cryst Eng Mater. 2021 May 13;77(Pt 3):357–64. doi: 10.1107/S2052520621003309 (PMC8182801; doi:10.1107/S2052520621003309)
Supplement: Supplementary file 3 [file b-77-00357-sup1.pdf]

Acta Cryst B
STRUCTURAL SCIENCE
CRYSTAL ENGINEERING
MATERIALS

Volume 77 (2021)

Supporting information for article:

A survey of thermal expansion coefficients for organic molecular crystals in the Cambridge Structural Database

Andrew D. Bond

Routines for extracting the volumetric and principal expansion coefficients were implemented using Python (v3), for compatibility with the CSD Python API. The methodology largely follows the implementation of *PASCal* (Cliffe & Goodwin, 2012), except where highlighted below.

S1. Extracting the volumetric expansion coefficient

Since the unit-cell volume is directly available for each CSD entry, the volumetric expansion coefficient can be extracted from the linear LS fit of *V* vs *T*. The gradient defines $\Delta V/\Delta T$, but it is necessary to choose a reference value for *V*. For consistency across the large range of structure families, the reference volume is extrapolated to 298 K, as calculated by the parameters of the LS fit.

α_V = 1E6 × (LS gradient / V (298 K)) ppm K^{-1} for the plot of *V* vs *T*.

The α_V value calculated by *PASCal* is based on an LS fit of ***T* vs *V*** (since this enables weights to be applied conveniently to the *T* values). For good linear fits, there is little difference between a fit of *V* vs *T* and *T* vs *V*. For data sets deviating significantly from linearity, however, the value obtained from *T* vs *V* can differ substantially from that obtained using *V* vs *T*. **A consistent approach with *T* as the independent variable is applied in this paper and recommended for comparison of α_V to the presented distributions.** See ABELAU below for an example.

S2. Calculating the strain tensor

Calculation of the strain tensor follows the description given for *PASCal*. At the heart of the method is the transformation matrix to yield Cartesian axes from the crystal axes, which depends on the convention chosen. Schlenker (1978), *PASCal* and the Bilbao Crystallographic Server all choose the Institute of Radio Engineers (IRE) convention: *z*(cart) parallel to crystal *c*, *x*(cart) parallel to crystal *a**, and *y*(cart) perpendicular to *x*(cart) and *z*(cart). The same convention was implemented in the Python code, and the resulting strain tensors were validated against the *STRAIN* module of the Bilbao server.

S3. Calculating the principal expansion coefficients

The three principal strains, $\Delta L/L$, are obtained as the eigenvalues of the strain tensors. For each data point above the minimum temperature, the strain tensor is calculated relative to the lowest temperature, then a linear LS fit is applied. As for *PASCal*, the eigenvalues at each step are sorted by magnitude and assumed to be in the same sequence through the range. The resulting α_L values are relative to *L* at the minimum temperature in the supplied range. For consistency with the approach applied to the volume fit, the values are re-scaled to refer to *L* at *T* = 298 K.

α_L = 1E6 × LS gradient / (1 + $\Delta L/L$ (298 K)) ppm K^{-1} for the plot of each eigenvalue vs *T*.

S4. Calculating errors

Standard uncertainties are calculated using heteroscedasticity-consistent standard errors, defined at: https://en.wikipedia.org/wiki/Heteroscedasticity-consistent_standard_errors. For α_V, the quoted standard uncertainty is the standard error on the gradient of V vs T, divided by the reference volume extrapolated to 298 K. For α_L, the quoted standard uncertainty is the standard error on the gradient of $\Delta L/L$ vs T, divided by the reference value of $\Delta L/L$ extrapolated to 298 K.

S5. Fitting of the distributions

Histograms were produced using *EXCEL*, with bin ranges chosen to provide a smooth representation of the distribution. Continuous distributions were fitted to the derived histogram values using the *SOLVER* within *EXCEL*. For the volumetric coefficient, a normal distribution was applied. Three parameters (scale, mean, su) were optimised so as to minimise the sum of the squared differences between the normal distribution and the value in each histogram bin. The temperature points for the fit were taken to be the midpoint of each bin. For the principal coefficients and the anisotropy measure, the histogram was initially fitted using a skew normal distribution (https://en.wikipedia.org/wiki/Skew_normal_distribution), defined in *EXCEL* as follows:

scale * NORM.DIST(*T*, mean, su, FALSE) * NORM.DIST(alpha**T*, alpha*mean, su, TRUE)

The first term is a symmetrical normal distribution and the second term is a cumulative normal distribution, multiplied by the skew parameter alpha. Four parameters (scale, mean, su, alpha) were optimised so as to minimise the sum of the squared differences between the normal distribution and the value in each histogram bin. The resulting continuous skew normal distribution was then approximated by two half normal distributions, defined with a common mean, but individual standard deviations and a single scale parameter linked by the ratio (su(R)/su(L)). The four parameters defining these two half normal distributions were optimised so as to minimise the squared differences relative to the continuous skew normal distribution over the full range of the plot, with this sum of squares including the lower half of the left distribution and the upper half of the right distribution. The values quoted in the paper are rounded to integers, to avoid any false indication of precision.

S6. Chemical diagrams for the structures referred to in the text

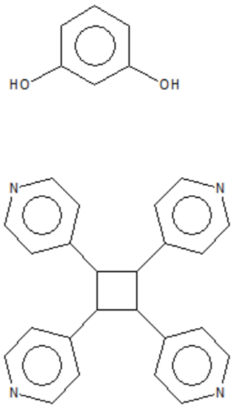	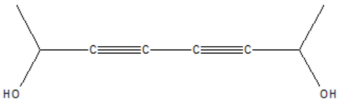	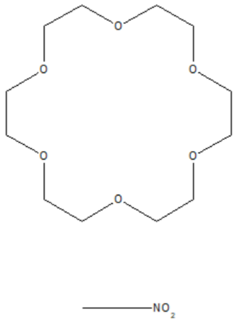
ABELAU	AHEJAZ	BIJWAS
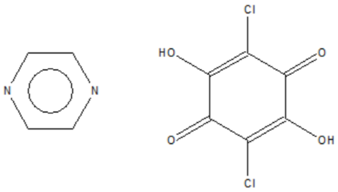	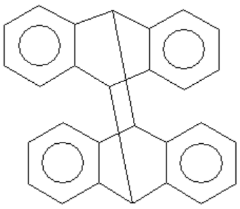	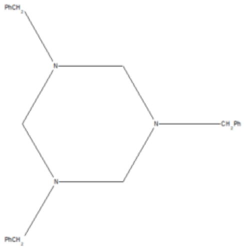
BOQHOE	DPANTH	FOCGOT
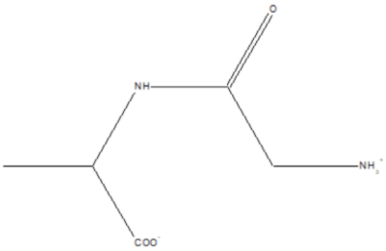	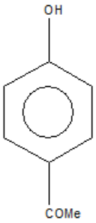	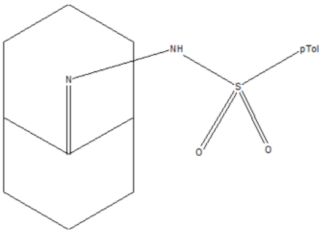
GLYALB	HACTPH	JETRIJ
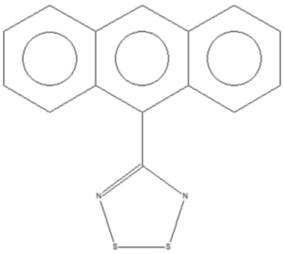	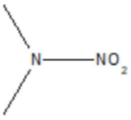	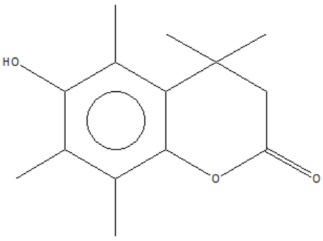
JOGVEJ	METNAM	MEZKEH

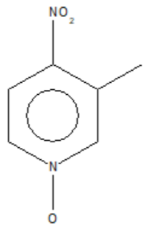	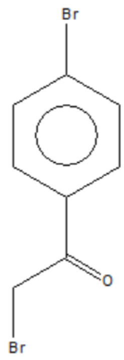	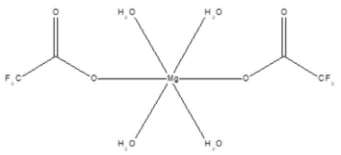
MNPYDO	PBPACB	RALLAU
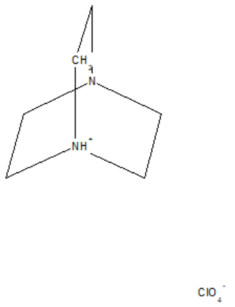	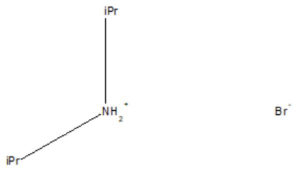	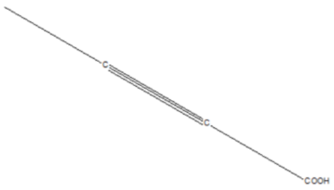
TEDAPC	TEJKUO	TETROL
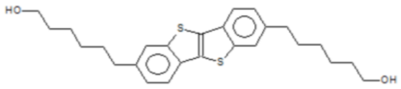	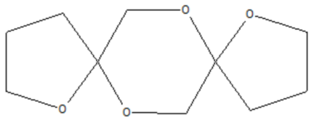	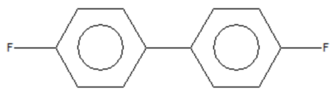
UROBUA	XIWREA	ZZZAOS
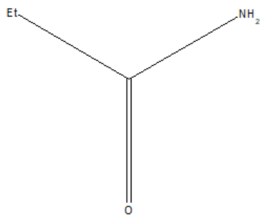		
ZZZKAY		

S7. Selected examples

S7.1. ABELAU (MacGillivray *et al.*, 2000; Hutchins *et al.*, 2018*b*)

Table S1 This is noted in the text to be an example of a family containing a significant outlier at 173 K.

Refcode	*T* (K)	*a* (Å)	*b* (Å)	*c* (Å)	α (°)	β (°)	γ (°)	*V* (Å^3)
ABELAU	173	9.749	11.367	14.273	111.668	90.000	90.000	1469.926
ABELAU01	190	9.749	11.324	14.245	111.822	90.000	90.000	1459.897
ABELAU02	210	9.753	11.316	14.248	111.746	90.000	90.000	1460.435
ABELAU03	230	9.770	11.322	14.267	111.713	90.000	90.000	1466.124
ABELAU04	250	9.776	11.314	14.269	111.658	90.000	90.000	1466.780
ABELAU05	270	9.789	11.314	14.283	111.607	90.000	90.000	1470.797
ABELAU06	291	9.802	11.312	14.292	111.539	90.000	90.000	1474.028

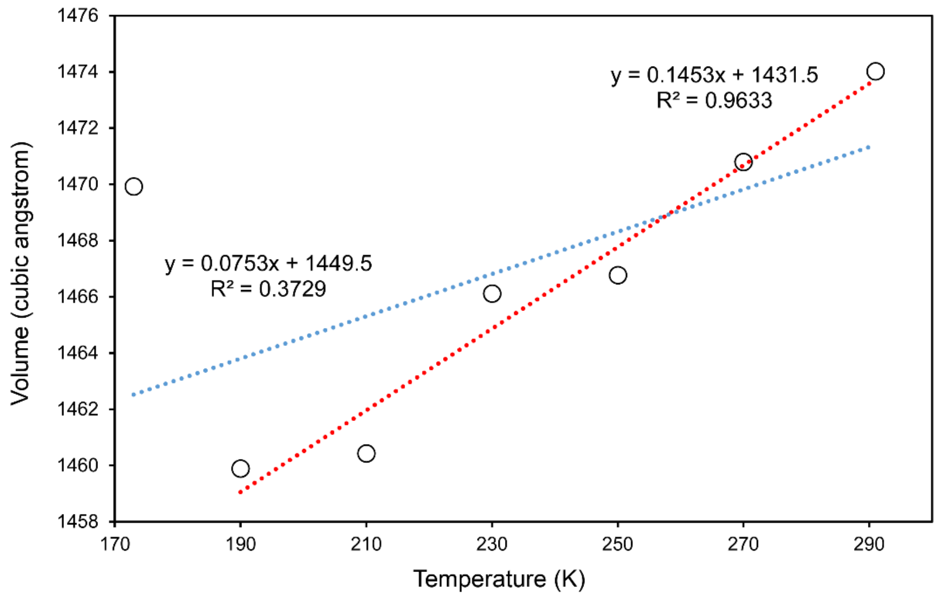

Figure S1 [Blue line shows the LS fit to all data points; red line shows LS fit excluding ABELAU at 173 K]

Table S2 **Python code**: including all structures in the fit of *V* vs *T* (R^2 = 0.3729):

Reporting *T*	$\alpha_L(1)$ (ppm K^{-1})	$\alpha_L(2)$ (ppm K^{-1})	$\alpha_L(3)$ (ppm K^{-1})	$\Sigma\alpha_L$	α_V (ppm K^{-1})
0	–23 (20)	26 (12)	49 (5)	52	52 (37)
173	–23 (20)	26 (12)	49 (5)	52	52 (37)
298	–23 (20)	26 (11)	49 (5)	52	51 (36)

Python code: omitting ABELAU from the fit of *V* vs *T* (R^2 = 0.9633):

**Reporting *T***	**α_L(1) (ppm K^{-1})**	**α_L(2) (ppm K^{-1})**	**α_L(3) (ppm K^{-1})**	**$\Sigma\alpha_L$**	**α_V (ppm K^{-1})**
0	–12 (3)	56 (4)	57 (2)	101	102 (8)
190	–12 (3)	56 (4)	56 (2)	100	100 (8)
298	–12 (3)	55 (4)	56 (2)	99	99 (8)

PASCal (unit weights on *T*): including ABELAU:

**Reporting *T***	**α_L(1) (ppm K^{-1})**	**α_L(2) (ppm K^{-1})**	**α_L(3) (ppm K^{-1})**	**$\Sigma\alpha_L$**	**α_V (ppm K^{-1})**
173	–23 (17)	26 (10)	49 (4)	52	136 (51)

For this case, with the poor linear fit to all of the points, the volumetric coefficient derived from a fit of *T* vs *V* is significantly different from the sum of the principal coefficients.

PASCal (unit weights on T): omitting ABELAU:

**Reporting *T***	**α_L(1) (ppm K^{-1})**	**α_L(2) (ppm K^{-1})**	**α_L(3) (ppm K^{-1})**	**$\Sigma\alpha_L$**	**α_V (ppm K^{-1})**
190	–13 (2)	55 (3)	56 (1)	98	102 (7)

S7.2. MNYPDO

Table S3 The largest identified structure family, and noted in the text to contain a clear outlier at 296 K. The orthorhombic structure permits an independent check on $\Delta L/L$ calculations and extrapolation of α_L to 298 K, by a direct plot of *L* vs *T*.

Refcode	*T* (K)	*a* (Å)	*b* (Å)	*c* (Å)	α (°)	β (°)	γ (°)	*V* (Å^3)
MNPYDO01	106	5.135	6.094	20.890	90.000	90.000	90.000	653.704
MNPYDO04	120	5.133	6.109	20.953	90.000	90.000	90.000	657.090
MNPYDO05	135	5.134	6.111	20.992	90.000	90.000	90.000	658.589
MNPYDO06	150	5.134	6.113	21.022	90.000	90.000	90.000	659.795
MNPYDO07	165	5.134	6.114	21.059	90.000	90.000	90.000	661.102
MNPYDO08	180	5.135	6.116	21.098	90.000	90.000	90.000	662.587
MNPYDO09	195	5.136	6.116	21.132	90.000	90.000	90.000	663.867
MNPYDO10	210	5.137	6.119	21.172	90.000	90.000	90.000	665.542
MNPYDO11	225	5.138	6.120	21.216	90.000	90.000	90.000	667.173
MNPYDO12	240	5.139	6.122	21.260	90.000	90.000	90.000	668.856
MNPYDO30	255	5.141	6.123	21.300	90.000	90.000	90.000	670.507
MNPYDO13	270	5.143	6.126	21.343	90.000	90.000	90.000	672.455
MNPYDO14	285	5.146	6.128	21.383	90.000	90.000	90.000	674.310
MNPYDO26	296	4.986	6.001	20.343	90.000	90.000	90.000	608.622

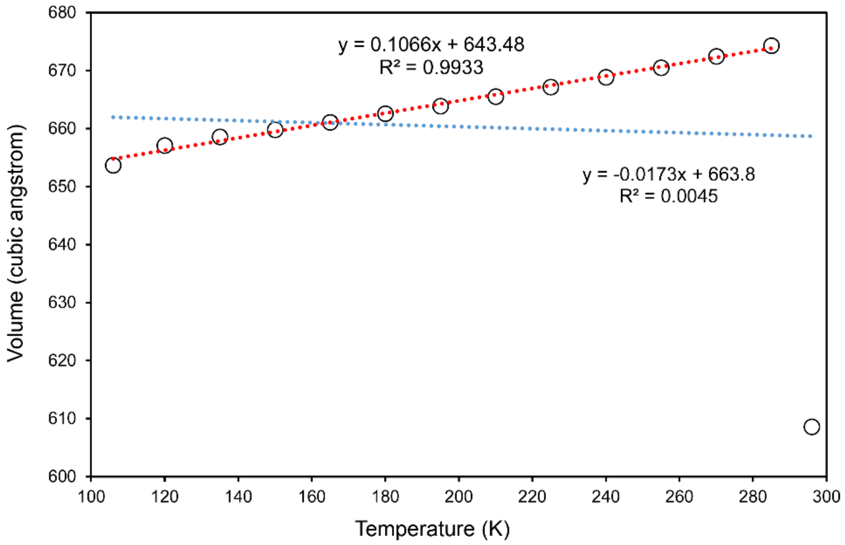

Figure S2 [Blue line shows the LS fit to all data points; red line shows LS fit excluding MNPYDO26 at 296 K]

Table S4 <u>**Python code**</u>: including all structures in the fit of V vs T (R^2 = 0.0045):

Reporting T	$\alpha_L(1)$ (ppm K^{-1})	$\alpha_L(2)$ (ppm K^{-1})	$\alpha_L(3)$ (ppm K^{-1})	$\Sigma\alpha_L$	α_V (ppm K^{-1})
0	–45 (50)	–37 (53)	53 (66)	–29	–26 (164)
106	–45 (51)	–37 (53)	52 (66)	–30	–26 (165)
298	–46 (51)	–37 (53)	52 (65)	–31	–27 (166)

<u>**Python code**</u>: omitting MNPYDO26 from the fit of V vs T (R^2 = 0.9933):

**Reporting T**	**$\alpha_L(1)$ (ppm K^{-1})**	**$\alpha_L(2)$ (ppm K^{-1})**	**$\alpha_L(3)$ (ppm K^{-1})**	**$\Sigma\alpha_L$**	**α_V (ppm K^{-1})**
0	12 (2)	23 (4)	129 (2)	164	166 (5)
106	12 (2)	23 (4)	127 (2)	162	163 (5)
298	12 (2)	23 (4)	124 (2)	159	158 (5)

<u>**PASCal (unit weights on T)**</u>: omitting MNPYDO26:

**Reporting T**	**$\alpha_L(1)$ (ppm K^{-1})**	**$\alpha_L(2)$ (ppm K^{-1})**	**$\alpha_L(3)$ (ppm K^{-1})**	**$\Sigma\alpha_L$**	**α_V (ppm K^{-1})**
106	12 (2)	23 (4)	127 (2)	162	164 (5)

<u>**Check using a linear fits of V vs T and L vs T (calculations in *EXCEL*):**</u>

**T (K)**	**a (Å)**	**b (Å)**	**c (Å)**	**V (Å^3)**	**LS fit (a)**	**LS fit (b)**	**LS fit (c)**	**LS fit (V)**
106	5.135	6.094	20.890	653.704	5.1318	6.1039	20.9032	654.781
120	5.133	6.109	20.953	657.090	5.1326	6.1058	20.9404	656.273
135	5.134	6.111	20.992	658.589	5.1336	6.1079	20.9803	657.872
150	5.134	6.113	21.022	659.795	5.1345	6.1100	21.0202	659.471

165	5.134	6.114	21.059	661.102	5.1354	6.1121	21.0600	661.069
180	5.135	6.116	21.098	662.587	5.1364	6.1141	21.0999	662.668
195	5.136	6.116	21.132	663.867	5.1373	6.1162	21.1398	664.267
210	5.137	6.119	21.172	665.542	5.1382	6.1183	21.1797	665.866
225	5.138	6.120	21.216	667.173	5.1392	6.1204	21.2195	667.465
240	5.139	6.122	21.260	668.856	5.1401	6.1225	21.2594	669.063
255	5.141	6.123	21.300	670.507	5.1410	6.1246	21.2993	670.662
270	5.143	6.126	21.343	672.455	5.1420	6.1266	21.3392	672.261
285	5.146	6.128	21.383	674.310	5.1429	6.1287	21.3790	673.860

Gradients and extrapolated values for *L* and *V* based on the linear fits:

	a	***b***	***c***	***V***
Gradient * 1E6	62.12	138.89	2658.27	106586.3
Intercept (= value at 0 K)	5.1252	6.0891	20.6214	643.483
Value at 106 K	5.1318	6.1039	20.9032	654.781
Value at 298 K	5.1437	6.1305	21.4136	675.245

Calculated coefficients based directly on the LS fitted values of *L* and *V*:

T	**α_L(1) (ppm K^{-1})**		**α_L(2) (ppm K^{-1})**		**α_L(3) (ppm K^{-1})**		**α_V (ppm K^{-1})**	
**0**	62.12 / 5.1252	**12.12**	138.89 / 6.0891	**22.81**	2658.27 / 20.6214	**128.91**	106586.3 / 643.483	**165.64**
**106**	62.12 / 5.1318	**12.10**	138.89 / 6.1039	**22.75**	2658.27 / 20.9032	**127.17**	106586.3 / 654.781	**162.78**
**298**	62.12 / 5.1437	**12.08**	138.89 / 6.1305	**22.66**	2658.27 / 21.4136	**124.14**	106586.3 / 675.245	**157.85**

S7.3. AHEJAZ (Das *et al*., 2010)

Table S5 This example is noted in the text to be an exceptional case reported in the literature. The orthorhombic structure has principal axes aligned with the crystal axes, which permits independent checks on $\Delta L/L$ calculations, and extrapolation of α_L to 298 K, by a direct plot of *L* vs *T*.

Refcode	*T* (K)	*a* (Å)	*b* (Å)	*c* (Å)	α (°)	β (°)	γ (°)	*V* (Å^3)
AHEJAZ	225	4.616	11.699	15.191	90.000	90.000	90.000	820.335
AHEJAZ01	240	4.686	11.656	15.089	90.000	90.000	90.000	824.109
AHEJAZ02	255	4.743	11.638	15.025	90.000	90.000	90.000	829.313
AHEJAZ03	270	4.785	11.618	14.965	90.000	90.000	90.000	831.988
AHEJAZ04	285	4.819	11.615	14.938	90.000	90.000	90.000	836.189
AHEJAZ05	300	4.845	11.607	14.905	90.000	90.000	90.000	838.196
AHEJAZ06	315	4.868	11.607	18.883	90.000	90.000	90.000	840.984
AHEJAZ07	330	4.880	11.596	14.873	90.000	90.000	90.000	841.589

[Shaded rows are not used for the primary analysis (restricted to the range 90–300 K), but mentioned below when comparing to the results reported by Das *et al.*]

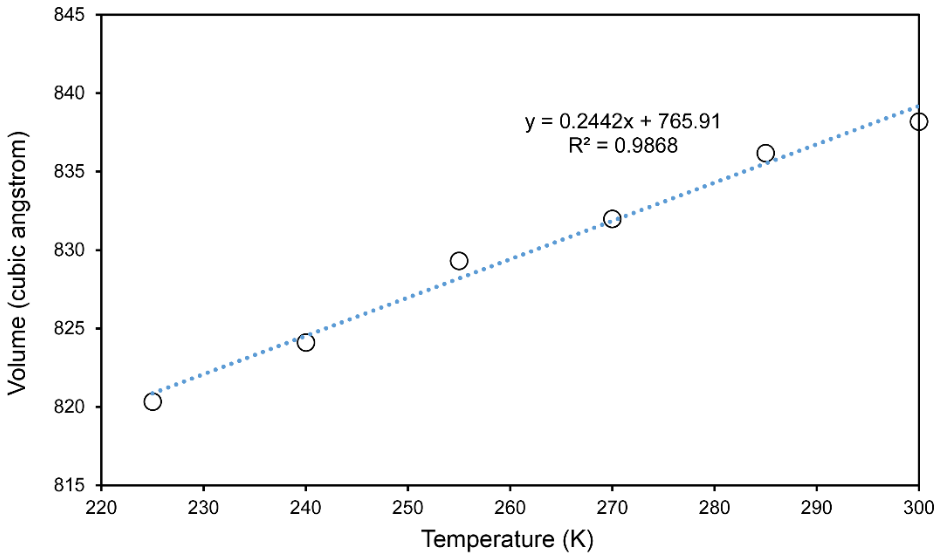

Figure S3 **Python code**: fit of V vs T (R^2 = 0.9868):

**Reporting *T***	**$\alpha_L(1)$ (ppm K^{-1})**	**$\alpha_L(2)$ (ppm K^{-1})**	**$\alpha_L(3)$ (ppm K^{-1})**	**$\Sigma\alpha_L$**	**α_V (ppm K^{-1})**
0	–231 (31)	–96 (21)	766 (79)	439	319 (19)
225	–244 (33)	–98 (21)	653 (68)	311	298 (18)
298	–249 (34)	–99 (22)	623 (64)	275	291 (17)

PASCal (unit weights on *T*):

**Reporting *T***	**$\alpha_L(1)$ (ppm K^{-1})**	**$\alpha_L(2)$ (ppm K^{-1})**	**$\alpha_L(3)$ (ppm K^{-1})**	**$\Sigma\alpha_L$**	**α_V (ppm K^{-1})**
225	–244 (27)	–98 (17)	654 (55)	312	300 (13)

Check using a linear fits of *V* vs *T* and *L* vs *T* (calculations in *EXCEL*):

***T* (K)**	***a* (Å)**	***b* (Å)**	***c* (Å)**	***V* (Å^3)**	**LS fit (a)**	**LS fit (b)**	**LS fit (c)**	**LS fit (*V*)**
225	4.616	11.699	15.191	820.335	4.6357	11.6819	15.1576	820.863
240	4.686	11.656	15.089	824.109	4.6810	11.6647	15.1021	824.527
255	4.743	11.638	15.025	829.313	4.7263	11.6474	15.0466	828.190
270	4.785	11.618	14.965	831.988	4.7717	11.6302	14.9911	831.853
285	4.819	11.615	14.938	836.189	4.8170	11.6130	14.9356	835.517
300	4.845	11.607	14.905	838.196	4.8623	11.5958	14.8800	839.180

Gradients and extrapolated values for L and V based on the linear fits:

	a	***B***	***c***	***V***
Gradient * 1E6	3020.95	–1148.57	–3700.95	244228.6

Intercept (= value at 0 K)	3.9560	11.9403	15.9903	765.912
Value at 225 K	4.6357	11.6819	15.1576	820.863
Value at 298 K	4.8562	11.5981	14.8874	838.692

Calculated coefficients based directly on the LS fitted values of L and V:

T	$\alpha_L(1)$ (ppm K^{-1})		$\alpha_L(2)$ (ppm K^{-1})		$\alpha_L(3)$ (ppm K^{-1})		α_V (ppm K^{-1})	
**0**	3020.95 / 3.9560	**763.6**	–1148.57 / 11.9403	**–96.2**	–3700.95 / 15.9903	**–231.5**	244228.6 / 765.912	**318.9**
**225**	3020.95 / 4.6357	**651.7**	–1148.57 / 11.6819	**–98.3**	–3700.95 / 15.1576	**–244.2**	244228.6 / 820.863	**297.5**
**298**	3020.95 / 4.8562	**622.1**	–1148.57 /11.5981	**–99.0**	–3700.95 / 14.8874	**–248.6**	244228.6 / 838.692	**291.2**

Comparison of values calculated in this paper to the values reported in Das *et al.*, 2010

The coefficients reported by Das *et al.* are calculated directly from the (orthorhombic) cell parameters, relative to the values at 330 K, *e.g.* $\alpha_L = 1/(330 - T) * (L(330) - L(T))$.

T (K)	α_a (ppm K^{-1})	α_b (ppm K^{-1})	α_c (ppm K^{-1})	α_V (ppm K^{-1})
225	514.9	–84.6	–203.6	241.0
240	441.7	–57.5	–161.4	231.0
255	374.3	–48.3	–136.3	194.9
270	322.4	–31.6	–103.1	190.1
285	274.6	–36.4	–97.1	142.6
300	237.0	–31.6	–71.7	134.7
315	155.7	–63.2	–44.8	47.5
330	0	0	0	0

This corresponds to taking a sequence of linear approximations between each lower temperature and the upper temperature of 330 K:

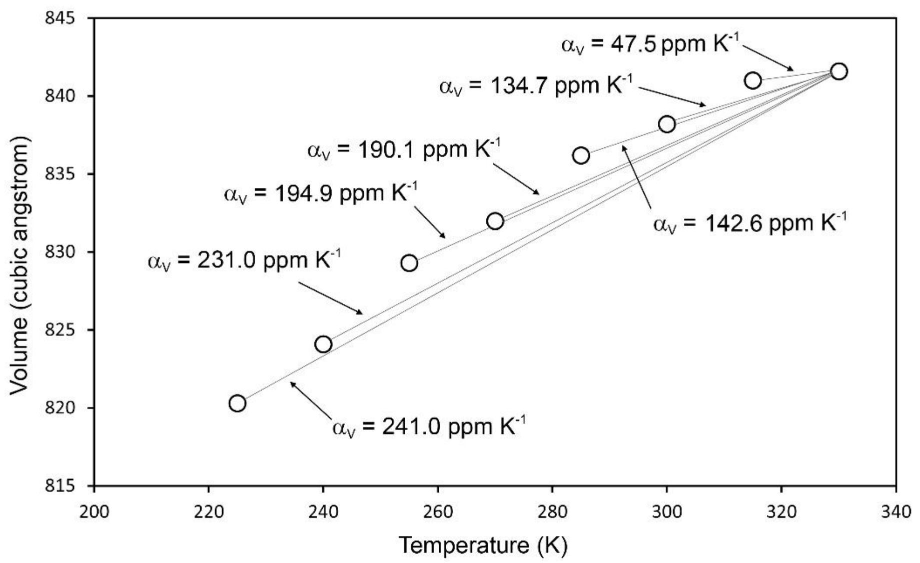

Figure S4 The results are then quoted as ranges: the expansion coefficients (ppm K^{-1}) of the axes lie in the range 156 < α_a < 515; –32 < α_b < –85 and –48 < α_c < –204 over the temperature range 225–330 K.

The approach in this current paper is to assume linearity of the plot of *V* (or *L*) against *T* (recalling that this is a necessity to deal with the vast majority of the data set extracted from the CSD), thereby producing a single fitted value for the gradient. Considering the full data range for AHEJAZ (225–330 K), this produces the following plots and coefficients:

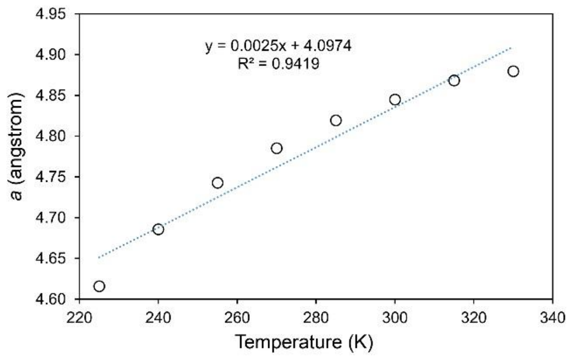

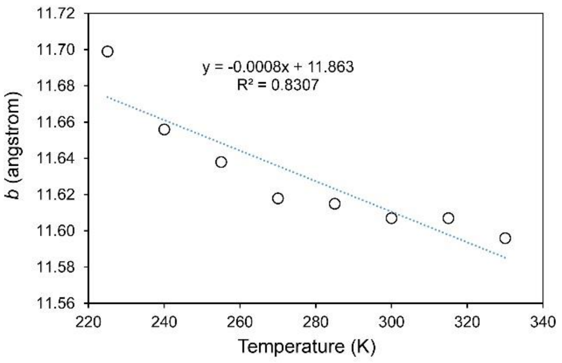

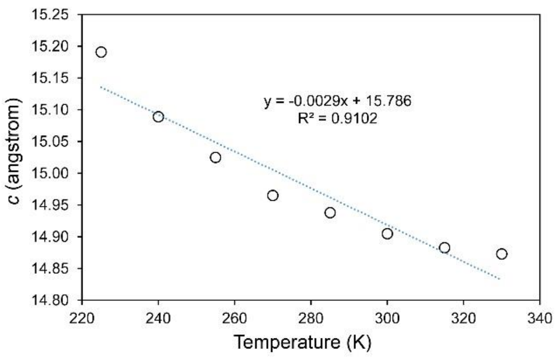

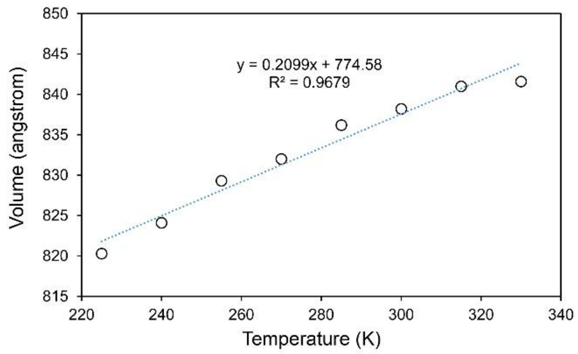

**Reference T**	**α_a (ppm K^{-1})**	**α_b (ppm K^{-1})**	**α_c (ppm K^{-1})**	**α_V (ppm K^{-1})**
298 K	509	–73	–194	251
330 K	501	–73	–195	249
